# Supplementary material for: Exploiting the Achilles’ Heel of Viral RNA Processing to Develop Novel Antivirals
Source: Viruses. 2024 Dec 31;17(1):54. doi: 10.3390/v17010054 (PMC11768839; doi:10.3390/v17010054)
Supplement: Supplementary file 1 [file viruses-17-00054-s001.zip › viruses-3366092-supplementary.pdf]

Supplementary Material

Table S1: Sequences of RTqPCR primers

Target	Forward Primer	Reverse Primer
IAV M1	ATCAGACATGAGAACAGAATGG	TGCCTAGCCTGACTAGCAACCTC
IAV M2	CGAGGTCGAAACGCCTATCAGAAAC	CCAATGATATTTGCGGCAATAGCGAG
IAV NS1	TGGAAAGCAGATAGTGGAGCG	GTAACGCGACGCAGGTACAGAG
IAV NS2	GCTCCAAATCAGAGTCTTGG	AGGCGTTCTCCTTGTAGGAG
IAV NP	AGGGTCGGTTGCTCACAAGTC	TTGAAGCAGTCTGAAAGGGTCTA
IAV M	CAAGACCAATYCTGTCACCTYTGAC	GCATTTTGGATAAAGCGTCTACG
HCoV 229E Total (N)	TATTATCTTGGCACAGGACC	TGAAGGATTCCGAGATTGAG
HCoV 229E Genomic	TGGGACTATCCTAAGTGGAT	GTACCACCAGGTTTAAAATAAA
SARS-CoV-2 Total (N)	CCTCTTCTCGTTCCTCATCA	CCTGGTCCCCAAAATTTCCT
SARS-CoV-2 Orf1ab (Genomic)	CCCTGTGGGTTTTACACTTAA	ACGATTGTGCATCAGCTGA
ACTIN	AGCTCATTGTAGAAGGTGTGG	GGCATGGGTCAGAAGGATTC
Adenovirus E1A	GGAATACGGGGGACCCAGA	ATTTTAGGACGGCGGGTAGG
Adenovirus E1B	ACATACTGACCCGCTGTTCC	AAACACCCCGTTCAGGTTCA
Adenovirus E2A	CGGTCTGGGCGTTAGGATAC	TGCAGATCTCCAACACCGAC
Adenovirus E2B	TTGTTGTGTAGGTACTCCGCC	CCTTGCGACTGTGACTGGTT
TBP	GATGCCTTATGGCACTGGAC	CCTTTGTTGCTCTTCCAAA

In vivo mouse studies

Studies investigating the effect of 5342191 administration in mice were performed by Nucro Technics Inc. under contract with Virocarb Inc. Compound was prepared in a vehicle consisting of 20% DMSO, 30% PEG400 and 50% phosphate buffered saline (PBS) (v/v/v) prior to administration. For examination of compound pharmacokinetics, mice were dosed either orally or intravenously and blood samples obtained for the measurement of the plasma concentrations of the test item. Two mice with blank plasma will also be collected for control purpose. For oral dosing, control or 5342191 were administered orally by gavage using a blunt tip gavage needle (20 - 22 G) attached to a syringe. For intravenous dosing, compound was administered via the lateral tail vein by a bolus injection over a 5-10 second period using a 26-23gauge needle attached to a syringe. Prior to dosing, the tail was warmed by the use of a heat lamp. Animals were observed at blood collection times. for changes in the skin and fur, eyes and mucous membranes, and also respiratory, circulatory, autonomic, and central nervous system, and somatomotor activity and behaviour pattern. For the purpose of collection of the samples, two mice per time point were first anesthetized with isoflurane and bled terminally by cardiac puncture. Each blood sample (approximately 0.8 mL) was collected into a tube containing an anticoagulant (K_2EDTA). Plasma was obtained from blood samples following centrifugation for 15 minutes at 3000 rpm and subsequently analyzed by Nucro-Technics' Bioanalytical Laboratory using a qualified method based on LC-MS/MS. Plasma concentration-time data will be analyzed by the non-compartmental method to obtain the pharmacokinetic parameters using validated Phoenix® WinNonlin® version 8.2 software (Certara Inc.).

For repeat dosing studies, mice were dosed with control and test items by the oral dosing route at dosing volumes 20 mL/kg, respectively, for 7 consecutive days. Compound was administered orally by gavage using a blunt tip plastic gavage needle (20 - 22 G) attached to a syringe. Prior to each individual dose between mice, the gavage needles were wiped clean with gauze. The volume administered to each animal was calculated based on the animals' body weight. Animals were observed closely for the first hour after dosing. Any animals exhibiting adverse events were examined by the veterinarian and continuously monitored until the clinical findings are resolved. Mortality checks and clinical observations were conducted daily. Moribund animals were euthanized for humane reasons at the discretion of the Clinical Veterinarian in consultation with the Study Director and the Sponsor (if possible). Animal body weights were recorded prior to the first dose (Day 1), and on the day of termination (Day 8).

Table S2: Summary of Body Weight Changes After Repeat Daily Oral 5342191 (C-191) Treatment

Treatment	Dose (mg/kg)	Mean Body Weight ± SD (g)		Mean Body Weight Changes Day 1-8 ± SD (g)	Animal number
		Day 1	Day 8		
Vehicle Control	0	24.7 ± 1.6	26.1 ± 2.0	1.4 ± 0.7	4
C-191	50	26.6 ± 3.7	27.7 ± 5.8	1.1 ± 0.6	2
	40	28.0 ± 1.7	27.6 ± 1.7	-0.4 ± 0.7	4
	30	26.1 ± 1.3	26.1 ± 0.9	0.1 ± 0.5	4
	10	27.8 ± 2.0	28.1 ± 1.8	0.4 ± 0.2	4

Table S3: Mean Plasma Concentrations upon Oral Dose of 5342191 (C-191) in Female Mice

Test Item	Dose (mg/kg)	Route	Time (hr)	N (Observed)	N	Average (µg/mL)	Min (µg/mL)	Max (µg/mL)
C-191	30	Oral	0.25	2	2	5.91	5.24	6.57
			0.5	2	2	4.04	3.51	4.57
			1	2	2	2.14	1.77	2.50
			2	2	2	1.91	1.84	1.97
			4	2	2	1.59	1.06	2.13
			8	2	2	2.12	1.92	2.33
			24	2	0	BLOQ	-	-

[illegible]

Table S4: Mean Group PK Parameters of Oral Dose 5342191 (C-191) in Female Mice

Test Item	Dose (mg/kg)	Route	C_{max} (µg/mL)	T_{max} (hr)	AUC_{last} (hr*µg /mL)	AUC_{INF} (hr*µg /mL)	AUC_{Extrap} (%)	Vz/F (mg/(µg/ mL)/kg)	Cl/F (mg/(hr*µg /mL)/kg)	MRT_{last} (hr)	K_{el} (1/hr)	$T_{1/2}$ (hr)	R^2
C-191	30	Oral	5.91	0.25	16.48	59.70	72	10.23	0.50	3.73	0.050*	14.11*	0.18*

[illegible]
